# Electroacupuncture for the prevention of acute gastrointestinal injury in patients with sepsis undergoing mechanical ventilation (EAGISM): a protocol for an open-label randomized controlled trial

**DOI:** 10.3389/fmed.2026.1695929

**Published:** 2026-03-05

**Authors:** Jian Xu, Ting-ting Xie, Jun-xuan Wu, Ding-wei Deng, Jing Huang, Yi Yu, Bo-jun Zheng, Ke-rui Wu, Jian Li

**Affiliations:** 1 Department of Critical Care Medicine, Guangdong Provincial Hospital of Chinese Medicine, The Second Affiliated Hospital of Guangzhou University of Chinese Medicine, Guangzhou, Guangdong, China; 2 Guangdong Provincial Key Laboratory of Research on Emergency in Traditional Chinese Medicine, Guangzhou, Guangdong, China; 3 The Second Clinical School of Medicine, Guangzhou University of Chinese Medicine, Guangzhou, Guangdong Province, China

**Keywords:** acupuncture, acute gastrointestinal injury, protocol, randomized controlled trial, sepsis

## Abstract

**Background:**

Septic acute gastrointestinal injury (S-AGI) plays a vital role in the initiation and progression of sepsis. Electroacupuncture (EA) is utilized for the treatment and prevention of S-AGI due to its positive impact on gastrointestinal function. However, there is a lack of rigorous trials examining the effectiveness and safety of EA in this context. Thus, a randomized controlled trial (RCT) at a single center has been designed to thoroughly assess the efficacy and safety of EA as an adjunctive therapy for preventing the incidence of S-AGI in patients with sepsis.

**Methods:**

A total of 200 mechanically ventilated patients aged ≥18 years with a clinical diagnosis of sepsis will be recruited from the Guangdong Provincial Hospital of Chinese Medicine in China. Patients will be randomly assigned in a 1:1 ratio to either the EA group or the control group. All patients in the control group will receive conventional drug therapy for S-AGI, while patients in the EA group will receive a combination of EA and conventional drug therapy. Only blood test assessors and statisticians will be blinded to the group assignment. The primary outcome is the incidence of S-AGI at day 10. Secondary outcomes include the severity grading of S-AGI, gastric retention, abdominal circumference, intra-abdominal pressure, frequency of bowel sounds, number of days tolerating the daily feeding target, proportion of patients receiving erythromycin and/or metoclopramide due to feeding intolerance, number of patients receiving one or more rectal laxatives due to constipation, plasma prealbumin levels, incidence rate of persistent inflammation, immunosuppression, and catabolism syndrome (PICS), number of days on invasive mechanical ventilation, length of stay in the intensive care unit (ICU), 7-day mortality rate, 28-day mortality rate, and adverse events (AEs).

**Discussion:**

The results of this study will provide substantial evidence regarding the efficacy of electroacupuncture in preventing S-AGI in patients with sepsis.

**Clinical trial registration:**

http://www.chictr.org.cn, Identifier ChiCTR2300078141.

## Introduction

### Background and rationale

Sepsis is defined as life-threatening organ dysfunction caused by a dysregulated host response to infection (1). Sepsis and septic shock are major causes of global morbidity and mortality (2), and data from high-income countries indicate that the annual incidence of sepsis is 31.5 million, with 19.4 million cases of severe sepsis contributing to 5.3 million potential deaths annually. Hospital mortality rates are reported to be 17% for sepsis and 26% for severe sepsis (3). There are limited comparable data from low- and middle-income countries, where the burden of sepsis may be even greater. Septic acute gastrointestinal injury (S-AGI) plays a key role in the initiation and progression of sepsis. Early-stage sepsis may cause intestinal barrier dysfunction, gastrointestinal motility disorder, and translocation of intestinal flora, leading to multiple organ dysfunction syndrome (MODS). Once MODS is initiated, it contributes to the development and worsening of gastrointestinal dysfunction, leading to increased hospitalization and mortality rates (4). According to existing literature, patients who were provided with preventive treatment for acute gastrointestinal injury (AGI) showed a significant increase in nutrient solution intake and a notable decrease in the duration of invasive mechanical ventilation compared to those who did not receive any preventive measures (5).

Treatments for S-AGI include early enteral nutrition, gastric motility drugs, acid inhibitors, and antimicrobial agents, which aim to protect the gastrointestinal mucosa, increase gastrointestinal motility, and improve intestinal flora (4, 6, 7). Despite the availability and application of S-AGI treatments, the case fatality rate for sepsis-3 is estimated to exceed 30%, corresponding to 700,437 deaths in China (8). Therefore, there is an urgent need to explore novel and supplemental treatments to improve clinical outcomes.

Growing evidence suggests that acupuncture alone, or in combination with Western medicine, can improve the clinical prognosis of S-AGI, including significant reductions in 28-day mortality and improvements in AGI grade and gastrointestinal function. A retrospective propensity score-matched cohort study conducted by our team evaluating the effects of electroacupuncture (EA) at “Zusanli” (ST36) and “Guanyuan” (RN4) on S-AGI demonstrated a 54% reduction in 28-day mortality among patients who received EA (9). Furthermore, in our recent randomized controlled trial (RCT), electroacupuncture combined with standard treatment improved clinical outcomes by regulating the immune system compared to standard treatment alone (10). Meng et al. conducted an RCT and reported that patients with S-AGI exhibited significant improvements in AGI grade and gastrointestinal function when acupuncture was used as an adjunctive therapy (11). However, findings regarding its impact on mortality have been inconsistent. Meng et al. found that the use of acupuncture as an adjuvant therapy decreased 28-day mortality in patients with S-AGI compared to standard treatment alone (11). However, another RCT reported no significant effect on mortality (12). Therefore, studies with a larger sample size are needed to clarify the effects of acupuncture on both gastrointestinal function and mortality in patients with sepsis.

### Objectives

The study aims to compare the preventive effectiveness of electroacupuncture combined with conventional treatment versus conventional treatment alone in patients with sepsis undergoing mechanical ventilation at risk of developing S-AGI.

## Methods

The EAGISM study—electroacupuncture for the prevention of AGI in patients with sepsis undergoing mechanical ventilation—follows the Declaration of Helsinki and was approved by the Ethics Committee of the Guangdong Provincial Hospital of Traditional Chinese Medicine (approval number: BF2023-228-01). The study was registered with the Chinese Clinical Trial Registry (identifier: ChiCTR2300078141) prior to the enrollment of the first participant. We followed the Standard Protocol Items: Recommendations for Interventional Trials guideline for the reporting of the study protocol (13).

### Study design

**Figure 1:**
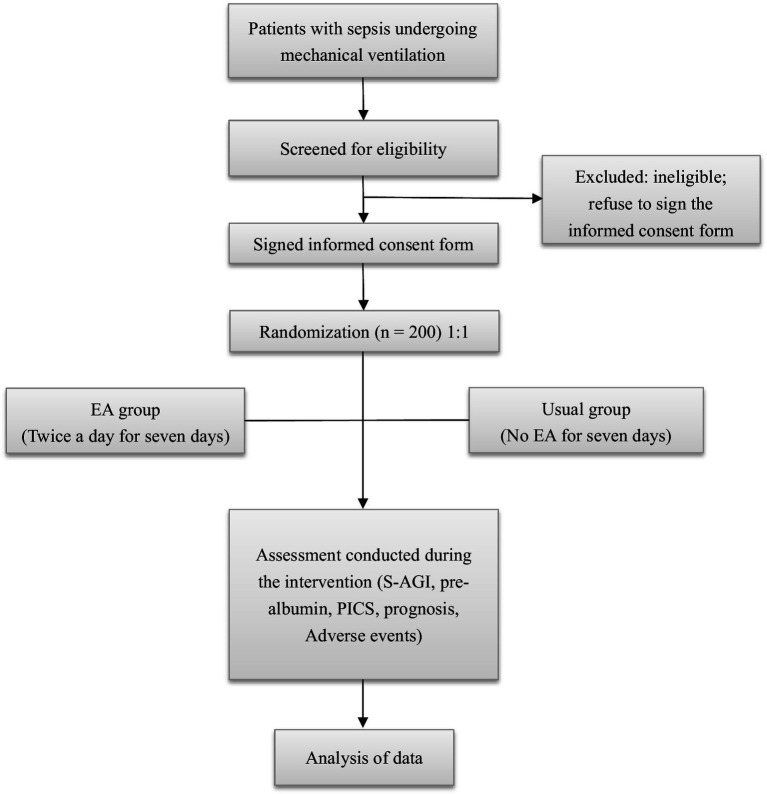
Flowchart of the trial procedure (EA, electroacupuncture; S-AGI, septic acute gastrointestinal injury; PICS, persistent inflammation, immunosuppression, and catabolism syndrome).

A single-center, two-arm, open-label randomized controlled trial will be conducted at the Guangdong Provincial Hospital of Traditional Chinese Medicine in China, focusing on patients with sepsis admitted to the intensive care unit (ICU). Participants will be randomly assigned to one of the two groups: electroacupuncture combined with conventional treatment or conventional treatment alone, administered during the initial 7-day period after randomization. Evaluations will be carried out at baseline and throughout the 28-day period following randomization. The trial design is depicted in Figure 1.

### Participants

Patients who meet the following criteria will be considered eligible: (1) age ≥18 years; (2) admission to the ICU specifically for sepsis, without any additional life-threatening injuries; (3) an expected duration of invasive mechanical ventilation of ≥72 h; (4) absence of S-AGI before enrollment, as no AGI standards have been met; (5) receipt of enteral nutrition via oral or nasogastric tube for 1 week or longer, as determined by the attending physician; and (6) provision of written informed consent by the patient or an authorized family member.

The diagnosis of sepsis is established based on the definition and diagnostic criteria outlined in the 2016 International Guidelines for the Management of Sepsis, which define sepsis as an imbalanced response to infection leading to dysfunction in vital organ systems, indicated by a sequential organ failure assessment (SOFA) score of ≥2. Accordingly, sepsis can be diagnosed when there is confirmed or suspected infection accompanied by a SOFA score of ≥2 (14).

Participants will be excluded if they meet any of the following criteria: (1) known or suspected acute gastrointestinal obstruction; (2) risk of gastrointestinal perforation; (3) history of peptic ulcer disease; (4) diagnosis of Crohn’s disease; (5) diagnosis of Ogilvie syndrome; (6) acute diverticulitis; (7) presence of infiltrative gastrointestinal tumors; (8) recurrent or advanced ovarian cancer with peritoneal metastasis; (9) history of abdominal trauma with potential risk of gastrointestinal perforation; (10) any other primary gastrointestinal diseases; (11) myocardial infarction within the past 6 months with evident clinical manifestations related to the congestive cardiovascular system and QT interval ≥500 ms; (12) family members opting for conservative treatment measures on behalf of the patient; (13) known pregnancy or breastfeeding; (14) severe liver cirrhosis classified as Child-Pugh grade C; (15) history of one or more life-threatening events; (16) participation in other clinical trials within the previous month, including ongoing trials, and (17) post-cardiac pacemaker surgery or presence of metal implants/prostheses in the body.

Our team of researchers will conduct a thorough assessment of potential candidates based on physical examinations and clinical evaluations. Prior to enrollment, eligible individuals will be requested to provide their consent by signing a confidentiality agreement, ensuring the protection of their personal information.

### Randomization

The SAS 9.2 software will be utilized to generate random numbers in accordance with the principle of randomization. To ensure allocation concealment, envelopes will be utilized for concealing the randomized sequence. Specifically, each random allocation will be placed in an opaque envelope with a corresponding code written on the exterior. Subsequently, these envelopes will be securely sealed and provided to the researchers. Upon meeting the inclusion criteria, research participants will be assigned a number, which corresponds to the opening of the designated envelope to implement the intervention based on the contained random concealment. Following a pre-established random allocation, patients will be randomly assigned to the electro-acupuncture group and the control group (1:1). Participant information will be collected using a case record form, and the EpiData software will be employed for the purpose of independent dual data entry. Subsequently, we will perform a thorough comparison between the two sets of data and cross-check any discrepancies to rectify input errors. The informed consent form will be obtained from each participant prior to randomization, under the supervision of the study coordinator at each site.

Only blood test assessors and statisticians will be blinded to the group assignment.

### Interventions

All patients will receive treatment for sepsis, as well as prevention and management of S-AGI, in accordance with established guidelines.

The standard treatment protocol for sepsis will adhere to conventional Western medical practices, in accordance with the “Surviving sepsis campaign: international guidelines for management of sepsis and septic shock (2021)” (15). This protocol will include management of the underlying condition, early and aggressive fluid resuscitation, administration of antibiotics, maintenance of hemodynamic stability, and support of other organ functions.

The enteral nutrition regimen and associated targets will be guided by the 2019 ASPEN/SCCM guidelines (16), which recommend a target caloric intake of 25–30 kcal/kg/day and a protein supply of 1.5 g/kg/day. The timing of initiation and dosage of enteral nutrition will be determined by the attending physician.

For the prevention and management of S-AGI, if gastrointestinal symptoms such as gastric retention or diarrhea occur, patients will receive Bifidobacterium supplementation at 0.84 g Bid orally. In cases of feeding intolerance, the attending physician will assess the need for erythromycin and/or metoclopramide. In addition, if constipation develops, one or more types of rectal laxatives may be utilized for treatment.

### Selection of acupuncture points

Previous clinical trials have confirmed that electroacupuncture at the Zusanli and Guanyuan acupoints can improve clinical outcomes in patients with S-AGI, possibly by improving gastrointestinal motility. A prospective controlled trial involving 60 sepsis patients showed that electroacupuncture at the Zusanli and Guanyuan acupoints improved clinical efficacy, potentially through mechanisms related to immune regulation (10). Another randomized controlled trial involving 55 sepsis patients found that abdominal acupuncture could improve symptoms of S-AGI by reducing gastrin and gastric secretions, thereby promoting gastrointestinal function recovery (17). Based on these findings, we chose the Zusanli and Guanyuan acupoints as the target acupuncture points for this research protocol.

### Methods of acupuncture in the EA group

Patients will be placed in a supine position for acupuncture treatment. Sterile, disposable stainless steel acupuncture needles (0.25 mm or 0.35 mm gauge, Hanyi Medical Instrument Co., Ltd., Beijing) will be gently inserted at the designated acupuncture points. For ST36, needles will be inserted perpendicularly to a depth of approximately 10–30 mm from the skin surface. For RN4, needles will be inserted obliquely to a depth of 10–30 mm at a 30-degree angle toward the patient’s feet, without lifting or thrusting. An electric stimulator (G6805-A EA apparatus; Huayi Industrial Co., Ltd., Shanghai, China) will be connected to the needle handles at the bilateral ST36 and RN4 points, delivering a continuous 5-Hz wave. The current intensity will be increased to a maximum level ranging from 1 to 5 mA, ensuring the absence of localized muscle twitching or any discomfort that might cause the patient to frown. The needles will be retained for 30 min. All acupuncturists will be licensed, have more than 3 years of clinical experience, and receive training on the specific study protocol.

Patients in the EA group will receive acupuncture treatment twice daily for 7 days. The first session will be conducted between 08:30 and 12:00, and the second session will be conducted between 14:00 and 17:00. A total of 14 acupuncture sessions will be conducted.

### Methods of acupuncture in the control group

Patients in the control group will receive only the conventional treatment described above, without acupuncture, for 7 days after randomization.

### Relevant concomitant care and interventions

In principle, within 7 days after randomization, all participants will not receive any other traditional Chinese medicine therapies, such as herbal medicine or moxibustion, except for the electroacupuncture specified in the protocol for the intervention group. Participants may receive other traditional Chinese medicine treatments prior to randomization, but not during the first 7 days following randomization. Receipt of any important interventions outside the study protocol should be documented in detail on the case record form.

### Outcomes

#### Primary outcome

The primary outcome is the 10-day incidence rate of S-AGI.

The occurrence of S-AGI is defined as the development of gastrointestinal symptoms in a patient who had no symptoms at baseline, with an AGI grade of 1 to 4 at the time of assessment.

#### Secondary outcomes

Secondary outcomes consist mainly of 16 indicators. It includes clinical and prognostic indicators (key secondary outcomes) and biochemical indicators (exploratory outcomes).

Clinical indicators include the following: (1) Average classification of the AGI grade, (2) gastric retention volume within 1 day, (3) abdominal circumference, (4) abdominal pressure, (5) frequency of bowel sounds, (6) number of days tolerating the daily feeding target, (7) proportion of patients receiving erythromycin and/or metoclopramide for feeding intolerance management, and (8) proportion of patients receiving one or more rectal laxatives for constipation management.

Biochemical indicators include the following: (1) Plasma concentration of motilin, (2) plasma concentration of gastrin, and (3) plasma concentration of prealbumin.

Prognostic indicators include the following: (1) Incidence of S-persistent inflammation, immunosuppression, and catabolism syndrome (PICS), (2) duration of invasive mechanical ventilation in days, (3) ICU length of stay in days, (4) mortality rate at day 7, and (5) mortality rate at day 28.

To assess gastrointestinal function, the AGI grade will be used (4). The AGI grade is widely used to evaluate gastrointestinal injury in patients with acute critical illness (4, 18). Evidence has shown that the AGI grading system is useful for determining the severity of gastrointestinal dysfunction/failure in sepsis (19). The specific criteria for AGI grading are provided in Supplementary File S1.

At baseline and on days 4, 8, and 10, AGI grading will be assessed, and gastric retention volume, abdominal circumference, abdominal pressure, and frequency of abdominal bowel sounds will be measured. The specific measurement procedures for these clinical indicators are detailed in Supplementary File S1. We will also record the gastric retention volume over the previous 24 h, the actual caloric intake from enteral nutrition, and any special treatments related to S-AGI, including the use of erythromycin and/or metoclopramide for feeding intolerance management or the use of one or more rectal laxatives for constipation management. In addition, we will collect venous blood samples from patients at baseline and on days 4, 8, and 10 to measure the plasma concentration of motilin and gastrin, which serve as indicators of gastrointestinal motility.

PICS was proposed by Gentile et al. to describe a series of clinical features characterized by persistent low-grade inflammation, immunosuppression, high catabolic metabolism, malnutrition, and muscle weakness in patients recovering from sepsis or severe trauma (20). Studies have shown that 43.1%–53.5% of sepsis patients develop PICS, and the mortality rate among sepsis patients with PICS is significantly higher than that among those without PICS (21). Therefore, PICS is considered a secondary trigger contributing to poor prognosis in sepsis patients (20). Based on this concept, in this trial, blood lymphocyte, serum C-reactive protein (CRP), serum albumin, and prealbumin levels on day 14 will be measured to evaluate the diagnostic criteria for PICS. At present, there are no unified standards for diagnosing PICS. Based on current literature (22, 23), PICS is defined by four criteria: ICU hospitalization ≥14 days; a continuous inflammatory response, defined as a CRP level >6 mg/L (normal range: 0–6 mg/L); immunosuppression, defined as a lymphocyte count <0.80 × 10^9^/L; and catabolism, defined as a serum albumin level <30 g/L or a prealbumin level <0.1 g/L.

In addition, the duration of invasive mechanical ventilation, ICU length of stay, mortality at day 7, mortality at day 28, and adverse events (AEs) will be recorded.

Outcome assessments will be performed by outcome assessors, who will receive standardized training in conducting interviews and performing measurements before study initiation and will follow a standard protocol. The schedule of measurements is presented in Table 1.

**Table 1:** Study schedule of the EAGISM trial.

Timepoint	D-1	D1	D2	D3	D4	D5	D6	D7	D8	D9	D10	D14	D28
Enrolment:	×												
Eligibility screen	×												
Informed consent	×												
Allocation	×												
Interventions:													
Acupuncture (EA group)		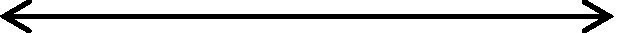											
Conventional treatment	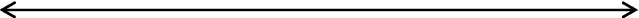												
Assessment:													
Baseline characteristics	×												
Important medical history	×												
Sepsis complications	×												
Site of infection	×												
Special treatment	×												
Lymphocyte count	×											×	
Albumin protein	×											×	
Prealbumin protein	×											×	
Gastrin	×				×				×		×		
Gastric	×				×				×		×		
AGI grade	×				×				×		×		
Gastric retention volume	×				×				×		×		
Abdominal circumference	×				×				×		×		
Abdominal pressure	×				×				×		×		
Abdominal bowel sounds	×				×				×		×		
Total caloric intake from EN	×	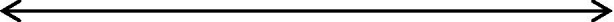											
Intake of protein	×	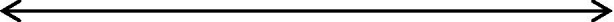											
Use of erythromycin/metoclopramide	×	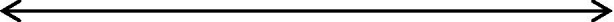											
Use of rectal laxatives	×	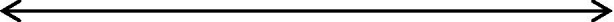											
Invasive mechanical ventilation	×	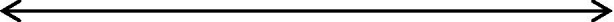											
Adverse events	×	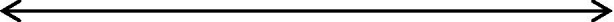										×	×
PICS												×	×
ICU length of stay	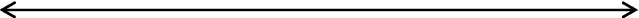												
Duration of invasive mechanical ventilation	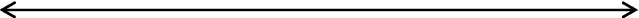												
Mortality status	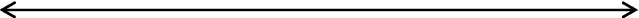												

### Safety assessment

While generally regarded as a safe therapeutic method with minimal risk of adverse effects (24), any adverse events (AEs) that occur during the study will be carefully evaluated, promptly addressed, and thoroughly recorded in the case report form (CRF). Adverse effects such as needle breakage, localized hematoma formation, bleeding, or severe local discomfort will be specifically attributed to the use of acupuncture, whereas those resulting from chemical or surgical interventions will not be considered related to acupuncture therapy. In the case of any serious adverse event (SAE) occurring in either group, it will be promptly reported to an independent ethics committee for thorough evaluation. This distinguished ethics committee is also responsible for conducting annual follow-up assessments of this research project.

### Quality control

To ensure study consistency, comprehensive training will be provided by the principal investigator (JX) to research staff regarding the study protocol, acupuncture manipulation techniques, outcome assessment methods, and participant follow-up procedures. Furthermore, it is important that a single research assistant or outcome assessor assumes responsibility for evaluating gastrointestinal function throughout the entire trial. Assessors will promptly collect and review all forms during each visit for outcome assessment and record the data in the CRF.

### Data management

The collected data will first be documented in the CRF by clinical research coordinators, then thoroughly reviewed by two trained clinical research associates, and finally entered into the EpiData software by a trained research assistant. This rigorous protocol ensures the utmost accuracy, authenticity, and traceability of the data. Furthermore, an independent data monitoring committee will be established to oversee and manage the quality of the data, ensuring complete impartiality without any conflicts of interest with the researchers or sponsors.

### Sample size

A parallel-design, randomized controlled trial methodology will be employed in the trial, with the primary outcome measure being the incidence of S-AGI. Based on clinical observations, the estimated incidence of S-AGI in the conventional treatment group is 70%. It is anticipated that electroacupuncture can potentially reduce the occurrence rate of S-AGI to 50%. With a significance level (*α*) of 0.05 and a power of 0.8, the sample size calculation yielded 94 participants per group. To account for potential loss to follow-up, we plan to include a total of 200 sepsis patients, with 100 patients allocated to each group. To account for uncertainties and maintain adequate power if the effect size is smaller than expected, sample sizes were also calculated as 163 participants per group for a 15% effect size and 356 participants per group for a 10% effect size.

### Statistical analysis

Statistical analysis will be performed using the SPSS software (version 29.0). For continuous data, a normality test will initially be conducted to assess distribution characteristics. If the data exhibit a normal distribution, descriptive statistics such as mean ± standard deviation (*SD*) will be used, and hypothesis testing will be carried out using a *t*-test. Conversely, if the data do not follow a normal distribution, descriptive statistics such as median and interquartile range (*IQR*) will be employed, and hypothesis testing will be performed using the *Mann–Whitney U* test. Categorical data will be described in terms of frequencies and percentages, with the chi-squared test used for hypothesis testing. The significance level for this study will be set at *α* = 0.05, with statistical significance defined as a *p*-value of <0.05. This trial includes 13 key secondary outcome indicators, covering both clinical and prognostic measures. To address multiplicity and control type I error, we applied the *Bonferroni* correction at both levels.

## Discussion

In our clinical research proposal, we focus on sepsis patients who require invasive mechanical ventilation. Invasive mechanical ventilation is common among sepsis patients admitted to the ICU, with studies indicating that approximately 21.38%–51.67% of sepsis cases necessitate this intervention (25, 26).

According to our clinical observations, several factors contribute to the increased susceptibility of mechanically ventilated sepsis patients to S-AGI. First, these individuals often exhibit a more severe clinical status, characterized by hypotension and multi-organ dysfunction that can compromise splanchnic blood flow and gastrointestinal integrity. Second, the use of sedatives and analgesics during mechanical ventilation can diminish gastrointestinal motility and impair the protective mucosal barrier, further predisposing patients to S-AGI. Moreover, the inflammatory response associated with sepsis can directly disrupt gut barrier function. A previous study reported that clinical ill patients who received preventive treatment for AGI exhibited a notably higher intake of the nutrient solution and a considerably shorter duration of invasive mechanical ventilation compared to those without any preventive measures (5).

S-AGI significantly impacts the prognosis of sepsis patients, increasing the risk of complications and mortality. Currently, there are no effective preventive strategies for S-AGI, underscoring the need for alternative interventions. Electroacupuncture has shown potential in preventing S-AGI; however, high-quality evidence from RCTs remains scarce. Therefore, it is crucial to conduct an RCT to establish the efficacy of electroacupuncture in preventing S-AGI, providing much-needed evidence to guide clinical practice and improve outcomes in sepsis patients.

In this clinical trial, we opt for an open-label design rather than using sham acupuncture as a control due to the lack of recognition and rigor associated with sham acupuncture methods. The current understanding in the field suggests that sham acupuncture does not adequately mimic the physiological effects of true acupuncture. Furthermore, sham acupuncture has several drawbacks, including the difficulty of effectively blinding both participants and clinicians. This can lead to biases in outcome reporting and subjective assessments, which can ultimately compromise the validity of the results (27–29). In addition, patients with sepsis requiring invasive mechanical ventilation usually require analgesia and sedation, remaining in a sedated state. Therefore, not using a sham acupuncture protocol in the control group will not affect outcome assessments. Consequently, we decided against employing sham acupuncture as a control in this RCT. Instead, the study will focus on evaluating the therapeutic effects of electroacupuncture compared to standard care, enabling a more direct assessment of its effectiveness in preventing S-AGI. This approach enhances the scientific credibility of the trial by minimizing bias and ensuring that the findings accurately reflect the intervention’s impact. By adopting an open-label design, our objective is to provide robust and dependable evidence that could inform clinical practices and improve treatment outcomes for sepsis patients at risk of AGI.

In open-label clinical studies, there is a high risk of selection, performance, and measurement bias. To minimize these biases, we will implement the following measures. First, to prevent selection bias, inclusion and exclusion criteria will be defined clearly to ensure baseline consistency among participants and reduce potential effects on outcomes. Randomization sequences will remain concealed until participants are confirmed eligible and enrolled. Second, to minimize performance bias, standardized intervention protocols and detailed manuals will be used in operations. Researchers will receive training from the project leader to ensure consistent implementation and assessment. Third, to mitigate measurement bias, outcome assessors and statisticians will be blinded to treatment assignments and data, respectively, until the analysis is complete. We will use an intention-to-treat approach to include data from all participants regardless of study completion, and we will report the reasons for, as well as the number of, missing data in each group.

This study will be the first large-sample randomized controlled trial to evaluate the efficacy of electroacupuncture in preventing S-AGI in patients with sepsis undergoing mechanical ventilation. We will assess the effects of electroacupuncture and compare the difference between the electroacupuncture and standard care groups in an adequately powered trial.
