# Supplementary material for: Electroacupuncture for the prevention of acute gastrointestinal injury in patients with sepsis undergoing mechanical ventilation (EAGISM): a protocol for an open-label randomized controlled trial
Source: Front Med (Lausanne). 2026 Mar 5;13:1695929. doi: 10.3389/fmed.2026.1695929 (PMC13001113; doi:10.3389/fmed.2026.1695929)
Supplement: Supplementary file 1 [file Table_1.docx]

**Supplementary File S1**

**Table s1 Definition of AGI grade**

| **AGI grade** | **Definiction** | **Examples** |
| --- | --- | --- |
| Grade I (risk of developing GI dysfunction or  failure) | The function of the GI tract is partially impaired, expressed as GI symptoms related to a known cause and perceived as transient, which expectedly has temporary and self-limiting nature. | Postoperative nausea and/or vomiting during the first days after abdominal surgery, postoperative absence of bowel sounds, diminished bowel motility in the early phase of shock. |
| Grade II (gastrointestinal dysfunction) | The GI tract is not able to perform digestion and absorption adequately to satisfy the nutrient and fluid requirements of the body. | Gastroparesis with high gastric residuals or reflux, paralysis of the lower GI tract, diarrhoea, intra-abdominal hypertension (IAH) grade I (intra-abdominal pressure (IAP)12–15 mmHg), visible blood in gastric contentor stool. Feeding intolerance is present if at least 20 kcal/kg BW/day via enteral route cannot bereached within 72 h of feeding attempt. |
| Grade III (gastrointestinal failure) | Loss of GI function, where restoration of GI function is not achieved despite interventions and the general condition is not improving. | Gastroparesis with high gastric residuals or reflux, paralysis of the lower GI tract, diarrhoea, intra-abdominal hypertension (IAH) grade I (intra-abdominal pressure (IAP) 12–15 mmHg), visible blood in gastric content or stool. Feeding intolerance is present if at least 20 kcal/kg /day via enteral route cannot be  reached within 72 h of feeding attempt. |
| Grade IV (gastrointestinal failure with severe  impact on distant organ function | AGI has pro-gressed to become directly and immediately life-threatening, with worsening of MODS and shock. | Bowel ischaemia with necrosis, GI bleeding leading to haemorrhagic shock, Ogilvie’s syndrome, abdominal compartment syndrome(ACS) requiring decompression. |

Tips: 1. Intra-abdominal hypertension: The intra abdominal pressure continuously or repeatedly rises to ≥ 12 mmHg.

2. Abdominal compartment syndrome (ACS): A continuous intra-abdominal pressure > 20 mmHg (with or without an abdominal perfusion pressure < 60 mmHg), along with the presence of new organ dysfunction/failure.

3. Grading of intra-abdominal hypertension: Grade I: Intra-abdominal pressure ranges from 12 to 15 mmHg; Grade II: Intra-abdominal pressure ranges from 16 to 20 mmHg; Grade III: Intra-abdominal pressure ranges from 21 to 25 mmHg; Grade IV: Intra-abdominal pressure > 25 mmHg.

4. High gastric residuals: It is defined as a single gastric fluid aspiration exceeding 250 ml or 500 ml/6 h.

**Measurement procedures for S-AGI-related indicators**

**Gastric retention:** Let the patient assume a semi-recumbent position, that is, raise the head of the bed to 45°. Connect a 50-milliliter syringe with the nasogastric tube and aspirate gastric juice to clarify the amount of gastric retention.

**Abdominal circumference:** Let the patient assume a supine position with both lower limbs extended naturally to fully expose abdomen. Employ a soft measuring tape to encircle the abdomen once, passing through the center of the umbilicus. During the measurement, make sure that the measuring tape contacts fully with the body, yet avoiding tightening it around the abdomen. Each measurement should be conducted at the same position.

**Intra-abdominal pressure:** The intra-abdominal pressure is indirectly measured by detecting the intra-vesical pressure. Patients should be maintained at supine position in a quiet state. Under strict aseptic conditions, a urinary catheter is inserted to empty bladder, and then a sensor device is connected. 25 ml of normal saline at a temperature of 37-40°C is injected into the bladder through the urinary catheter and allowed to stay for 30-60 seconds. A zero-calibration operation is performed at the mid - axillary line of the iliac crest, and the value at the end of expiration is recorded.

**Bowel sounds:** During auscultation, the patient should be positioned in a supine position to ensure full exposure of the abdomen. Auscultate three areas around the umbilicus and calculate the average number of bowel sounds. Record the number of bowel sound within one minute. It is essential to ensure that each measurement is taken at the same position.
